# Incisionless transcranial MR-guided focused ultrasound in essential tremor: cerebellothalamic tractotomy

**DOI:** 10.1186/s40349-016-0049-8

**Published:** 2016-02-13

**Authors:** Marc N. Gallay, David Moser, Franziska Rossi, Payam Pourtehrani, Anouk E. Magara, Milek Kowalski, Alexander Arnold, Daniel Jeanmonod

**Affiliations:** Sonimodul, Center for Ultrasound Functional Neurosurgery, Leopoldstrasse 1, CH-4500 Solothurn, Switzerland; Rodiag Diagnostics Centers, Leopoldstrasse 1, CH-4500 Solothurn, Switzerland; Praxisgemeinschaft für Neurologie, Thunstrasse 95, CH-3006 Bern, Switzerland; Privatklinik Obach, Leopoldstrasse 5, CH-4500 Solothurn, Switzerland

**Keywords:** Cerebellothalamic tractotomy, Deep brain stimulation, Essential tremor, Essential tremor rating scale, Functional neurosurgery, Incisionless transcranial MR-guided focused ultrasound, Thalamotomy

## Abstract

**Background:**

Already in the late 1960s and early 1970s, targeting of the “posterior subthalamic area (PSA)” was explored by different functional neurosurgical groups applying the radiofrequency (RF) technique to treat patients suffering from essential tremor (ET). Recent advances in magnetic resonance (MR)-guided focused ultrasound (MRgFUS) technology offer the possibility to perform thermocoagulation of the cerebellothalamic fiber tract in the PSA without brain penetration, allowing a strong reduction of the procedure-related risks and increased accuracy. We describe here the first results of the MRgFUS cerebellothalamic tractotomy (CTT).

**Methods:**

Twenty-one consecutive patients suffering from chronic (mean disease duration 29.9 years), therapy-resistant ET were treated with MRgFUS CTT. Three patients received bilateral treatment with a 1-year interval. Primary relief assessment indicators were the Essential Tremor Rating Scale (Fahn, Tolosa, and Marin) (ETRS) taken at follow-up (3 months to 2 years) with accent on the hand function subscores (HF16 for treated hand and HF32 for both hands) and handwriting. The evolution of seven patients with HF32 above 28 points over 32 (group 1) differentiated itself from the others’ (group 2) and was analyzed separately. Global tremor relief estimations were provided by the patients. Lesion reconstruction and measurement of targeting accuracy were done on 2-day post-treatment MR pictures for each CTT lesion.

**Results:**

The mean ETRS score for all patients was 57.6 ± 13.2 at baseline and 25.8 ± 17.6 at 1 year (*n* = 10). The HF16 score reduction was 92 % in group 2 at 3 months and stayed stable at 1 year (90 %). Group 1 showed only an improvement of 41 % at 3 months and 40 % at 1 year. Nevertheless, two patients of group 1 treated bilaterally had an HF16 score reduction of 75 and 88 % for the dominant hand at 1 year after the second side. The mean patient estimation of global tremor relief after CTT was 92 % at 2 days and 77 % at 1-year follow-up.

**Conclusions:**

CTT with MRgFUS was shown to be an effective and safe approach for patients with therapy-refractory essential tremor, combining neurological function sparing with precise targeting and the possibility to treat patients bilaterally.

## Background

Essential tremor (ET) is the most common pathological tremor in humans, frequently undiagnosed, often refractory to conservative treatments, and with the potential to cause severe disability [1–4]. In this context, a neurosurgical option has been considered [5, 6].

Already in the late 1960s and early 1970s, stereotactic targeting of the posterior subthalamic area (PSA) also named prelemniscal radiation or posterior zona incerta was explored worldwide by different functional neurosurgical groups [7–12] to treat ET, as an alternative to the ventral intermediate nucleus thalamotomy (Vim of Hassler, corresponding to the posterior part of the thalamic ventral lateral nucleus (VLp)) [13–16]. VLp thalamotomies were extensively performed from the 1960s to the 1990s [17–26]. The published results of the posterior subthalamic approach, taking into account technical limitations of their time, were highly promising.

We correlate this with the sparing of the thalamocortical network and thus a reduction of neurological motor and cognitive deficits, particularly desirable in the case of bilateral treatments. Such sparing of the thalamus makes all the more sense that the pathology of essential tremor lies in the cerebellar input to the motor thalamus.

In deep brain stimulation (DBS) nowadays, the target chosen for ET is most of the time the ventral portion of the VLp [27–33], but there is a growing interest in the PSA [33–45].

There is sound histological evidence that the so-called PSA, also named prelemniscal radiation by Hassler or field H by Forel, includes in fact the cerebellothalamic (or dentato-thalamic) fiber tract on its way to the VLp [14, 46]. The dentato-rubro-thalamic denomination should not be used in humans, as no rubro-thalamic connections have been reported in the literature. The only evidence for such connections was described in the cat [47], and they were absent in the monkey [48].

Recent clinicopathological data point to the cerebellar system in ET [2, 49]. Purkinje cell losses and changes in their different cell compartments (dendrites, body, axon, basket cell processes, and climbing fiber-Purkinje connections) have been described, supporting the possibility of reduced inhibition of the dentate nucleus and thus overactivation of the VLp.

The technology of incisionless transcranial magnetic resonance (MR)-guided focused ultrasound (MRgFUS) has already been successfully applied to thalamotomies in chronic neuropathic pain and essential tremor [6, 50–53] and to pallidothalamic tractotomies [54] in Parkinson’s disease, with a precision inside half a millimeter not obtainable by techniques implying brain penetration [55, 56].

This case series is the first to include patients operated for chronic therapy-refractory ET with MRgFUS CTT. A technical report of the CTT performed with radiofrequency ablation was published by Magnin et al. [57], and Ledermann et al. [58] addressed the neuropsychological outcome of this specific procedure.

## Methods

### Patients

Twenty-one consecutive patients suffering from chronic, therapy-resistant ET treated in our center with at least 3-months follow-up were included. The mean patient age was 69.1 (SD± 9.2), and the mean disease duration was 29.9 years (SD± 15). All patients had bilateral tremor, without any case of dominant head tremor. For detailed patient characteristics, see Table 1.

**Table 1:** Characteristics of the 21 patients with means ± SD

Gender	6F/15M
Age in years	69.1 ± 9.2
Baseline tremor score on ETRS (0 to 144)	57.6 ± 13.2
Duration of tremor in years	29.9 ± 15.0
Family history of tremor	19/21
Alcohol-responsive tremor	16/21
Bilateral treatment	3/21

The patients were seen by neurologists who ascertained the diagnosis and the resistance to drug treatment. They received beta blockers (*n* = 18), primidone (*n* = 10), topiramate (*n* = 3), octanol (*n* = 1), clonazepam (*n* = 3), gabapentin (*n* = 3), modafinil (*n* = 1), and baclofen (*n* = 1). They were evaluated by an internist as to contraindications to surgery. No patient took anticoagulant or antiaggregant drugs within 10 days before surgery. Blood workout was performed, and all patients had normal electrolytes and coagulation status.

The selection criteria for surgical treatment were as follows:

1. ET with postural and/or kinetic components reaching an intensity of at least 3 over 4
2. Tremor resistance to pharmacological treatment or appearance of side effects of drugs preventing their use
3. Absence of dementia
4. Strongly diminished quality of life

Preoperative assessment included an Essential Tremor Rating Scale (ETRS) (Fahn, Tolosa, and Marin Tremor Rating Scale) [59], full neurological status including a video recording, Montreal Cognitive Assessment (MoCA), and Hospital Anxiety and Depression Scale (HADS).

Primary relief assessment indicators were postoperative ETRS, hand function subscore (item 11–14 of ETRS, describing spiral and line drawings and pouring) presented for the targeted hand over 16 points (HF16) and for both hands over 32 points (HF32), handwriting (item 10 of ETRS), drawing of spirals, and estimation of global tremor relief by the patient (in percent). Spirals were drawn with both hands with and without support on table. The worst tremulous spirals were always used in pre- and postoperative scoring of ETRS.

The patients signed an informed consent form after having been fully instructed about the treatment, its results, and risks.

CTT was performed unilaterally in 18 and bilaterally in 3 patients. In bilateral treatments, it was performed first on the left, with a 1-year interval for the second side. In two patients (1 and 2), a complement of targeting was performed on the already operated side during the second treatment session.

Postoperative follow-ups were performed at 3 months and 1 year. For international patients (16 over 21), the 3-month assessment was performed per correspondence with video recordings and drawing of spirals.

### Focused ultrasound procedure

The procedures were performed in a 3-T MR imaging system (GE Discovery 750, GE Healthcare, Milwaukee, WI, USA) using the ExAblate Neuro device (InSightec, Haifa, Israel).

Targeting was performed using the stereotactic multiarchitectonic Morel *Atlas of the Human Thalamus and Basal Ganglia* [14]. Target coordinates of the cerebellothalamic tract were 5.0 mm posterior to the mid-commissural line in the anteroposterior (AP) direction, 8.0 mm lateral to the thalamo-ventricular border in the mediolateral (ML) direction, and 3 mm below the intercommissural plane.

**Fig. 1:**
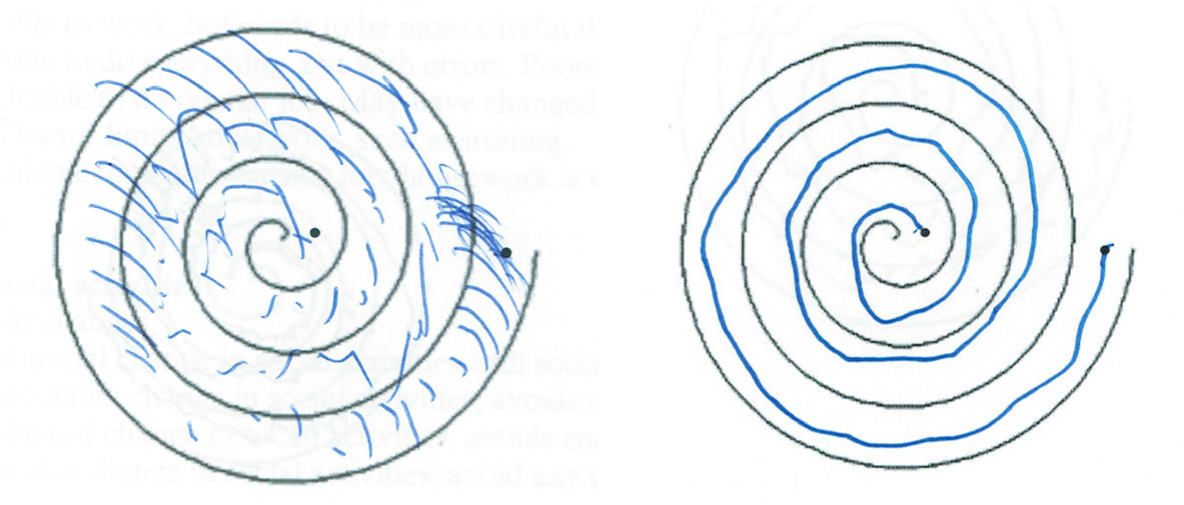
Spiral drawings of patient 17 reproduced from preoperative (*left*) and 2 days postoperative (*right*) assessments

**Fig. 2:**
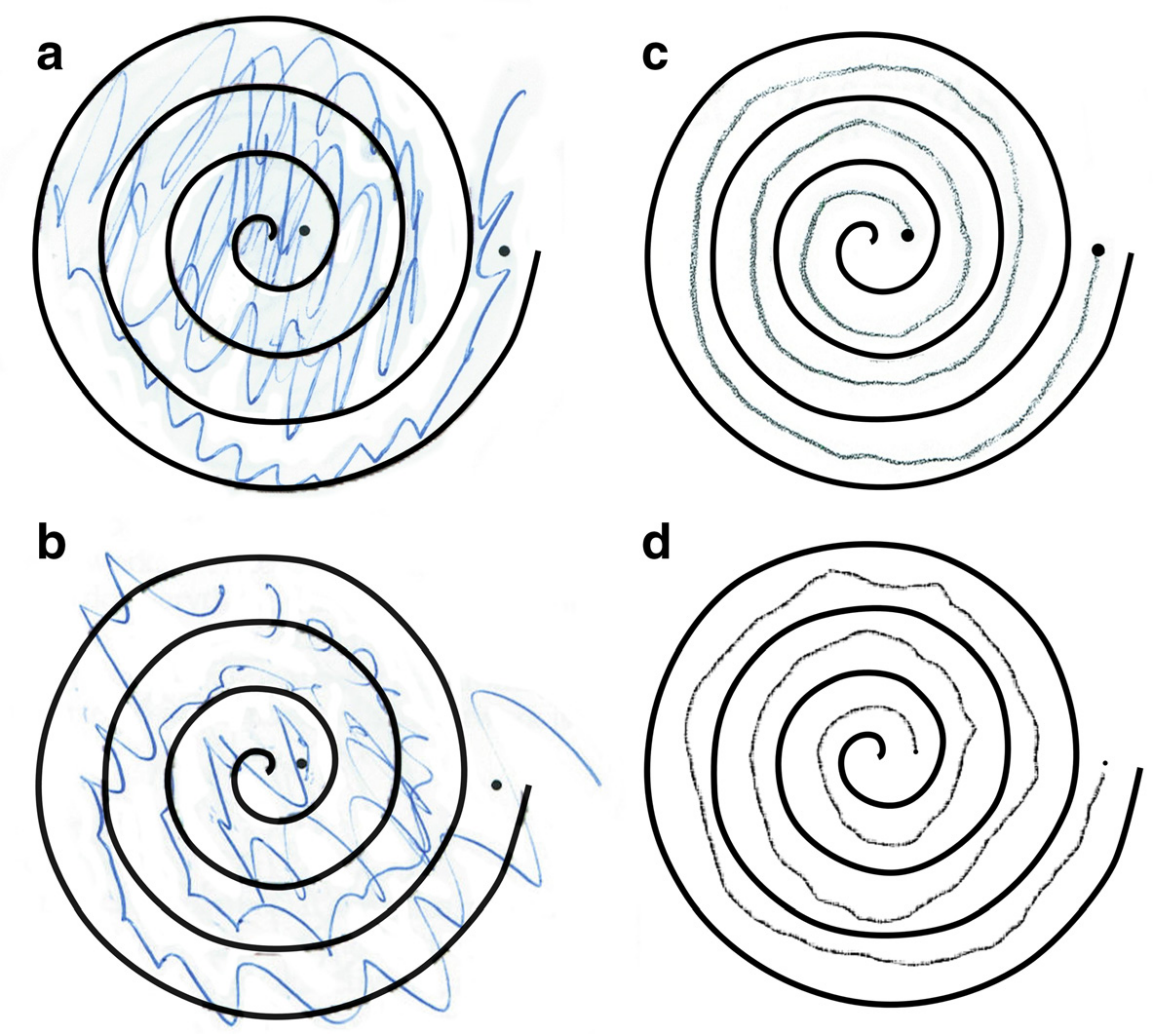
Reproduction of pre- and postoperative spirals in bilaterally treated patient 1. **a**, **b** Preoperative spirals drawn with the *right* (**a**) and *left* (**b**) hands. **c** Right hand, 2 years postoperative. **d** Left hand, 1 year after the second treatment

Shaving was performed 1 or 2 days before surgery and on the morning of the operation. The patients were fully awake during sonications. They received a mild anxiolytic (1.25–2.5 mg lorazepam) and gastric protection (pantoprazole 40 mg per os). Neurological integrity was controlled between every sonication. After reaching the end-temperature sonications, tremor tests including drawing of spirals were performed (Figs. 1 and 2). As described in Jeanmonod et al. and Magara et al. in the central lateral thalamotomies and pallidothalamic tractotomies [51, 54], respectively, low-power sonication rounds below the coagulation threshold (temperatures up to 45 °C) were guided by MR imaging and MR thermometry and allowed to assess and adjust the position of the thermal spot. They were followed by high-power sonications applied to achieve a final temperature at target between 54 and 60 °C. Those were repeated at least four times according to Magara et al. [54] to produce permanent and complete lesioning of the fiber bundle. This is in contrast to thalamic lesioning, which only needs a single temperature raise between 54 and 60 °C. Sufficient temperature was reached in every patient. The maximum applied energy was 30,800 J (mean 16,073, SD± 6,037), and the maximal power was 1250 W. The mean operation time from the stereotactic head frame fixation (Radionics, USA) to the frame ablation was 4.45 ± 1.1 h.

MR imaging was performed for co-registration preoperatively and at day 2 after surgery (Moser et al. 2012, 2013 and Magara et al. 2014) [54–56]. Target reconstruction was performed on the basis of the MR imaging at day 2 after surgery as previously described [55, 56] with comparison between desired target coordinates and coordinates of the geometric center of the lesions seen on the sagittal and axial T2-weighted images. As in Moser et al., targeting accuracy values were given for each of the three dimensions (ML, AP, and dorsoventral (DV)) in millimeters as well as 3D vector accuracy.

Statistical analysis was performed with Microsoft Excel and XL Toolbox for Excel, using *t* test, regression analysis and Levene’s test of equal variance. Statistical significance was fixed at *p* < 0.05.

## Results

### Clinical results

Clinical results are summarized in Table 2. The mean ETRS baseline score for all patients was 57.6 ± 13.2. At 1-year follow-up, the mean ETRS score was 25.8 ± 17.6 (*n* = 10), corresponding to a global ETRS score reduction of 55 %. The review of the individual ETRS baseline scores revealed strong differences between patients, and high preoperative scores seemed to be associated with less good postoperative results. This led us to search for the most appropriate predictor(s) of high postoperative tremor relief rates and for indicators for separation of patients in two groups. Regression analyses showed two significant predictors for functional improvement in the targeted hand with a *p* < 0.01: the preoperative ETRS (*r*^2^ = 0.32, *F* = 8.75, and *p* < 0.01) and HF32 (*r*^2^ = 0.34, *F* = 9.35, *p* < 0.01). The higher the preoperative score, the lower the percentage of improvement of the dominant hand score.

**Table 2:** The treatment characteristics include: ETRS, drawing of spirals, handwriting, gait instability, HADS, MoCA and hand function subscores (HF16 and HF32) at baseline (B), 2 days after the procedure (2d), 3 months (3m), and the last follow-up (F) ranging from 1 year (1y) to 2 years (2y) and in percent of improvement (%i)

			ETRS (B/3m/1y)	Spiral (B/F/%i)	Handwriting (B/F/%i)	Gait instability (B/2d/F)	HADS (B/2d)	MoCA (B/2d)		HF32 (B/2d)	HF16 (B/2d/%i/3m/%i/1y/%i)	HF16 (2d/1y after the second side/%i)
Group 1	1		77/–/52	4/1/75	4/0/100	0/0/0	14/8	29/30		32/20	16/4/75/8/50/9/44	2.5/4/75
	2		65/41.5/18	4/0.5/87.5	3/0.5/83	1.5/1.5/2.5	10/22	29/29		31/19	16/3/81/4/78/4/75	3.5/2/88
	3		55/53/56	4/3/25	4/3/25	1/1/0.5	5/7	25/26		29/21	13/5/62/13/0/13/0	–/–/–
	4		84/61/–	4/4/0	4/3/25	3/3/2	22/21	26/27		32/24	16/8/50/11/31/–/–	–/–/–
	5		70/–/–	4/1/75	3/0/100	1.5/1.5/–	16/–	25/25		32/17	16/1/94/5/69/–/–	–/–/–
	6		77/–/–	4/4/0	3.5/3.5/0	2/2/–	–/–	24/26		30/24	14/9/36/15/0/–/–	–/–/–
	7		70/68/–	4/1.5/62.5	4/3/25	2.5/2.5/3	15/9	26/29		32/23	16/7/56/7/56/–/–	–/–/–
Mean			71.1/55.9/42	4/2.1/46.4	3.6/1.9/51.2	1.6/1.6/1.6	13.7/13.4		26.3/27.4	31.1/21.1	15.3/5.3/64.8/9/40.6/8.7/39.6	3/3/81.3
SD±			9.4/11.4/20.9	0/1.5/–	0.5/1.6/–	1/1/1.3	5.8/7.4		2/1.9	1.2/2.7	1.3/2.9/–/4.1/–/4.5/–	0.7/1.4/–
Group 2		8	61/–/–	2/0/100	4/0/100	0/0/0	7/–		28/30	24/14	10/0/100/0/100/0/100	
		9	49/20/16	3/1/66.7	4/2.5/38	0/0/0	3/1		28/28	17/4	13/1/92/4/69/2/85	
		10	53/20/22	2.5/0/100	1/0/100	0/0/0	3/2		28/30	18/11	7/0/100/0/100/1/86	
		11	67/–/37	3/0.5/83	2.5/0/100	0/0/0.5	7/5		29/29	27/3	14/1/93/3/79/3/79	
		12	61/–/–	3/0/100	2/0/100	0/0/0	12/–		30/30	25/15	12/2/83/0/100/–/–	
		13	45/–/11	2.5/0/100	2/0/100	0.5/1/0.5	8/6		29/29	17/4	7/0/100/0/100/0/100	
		14	50/25/11.5	3.5/0/100	4/1.5/63	2/2.5/2	22/18		20/25	21/10	12/1/92/1/92/0/100	
		15	46/–/–	4/0/100	2/0/100	0/0/0	17/–		27/29	17/4	13/0/100/0/100/–/–	
		16	38/–/11.5	3/0.5/83	2.5/0/100	0/1/0	2/1		29/30	16.5/4	12.5/0/100/1/92/1/92	
		17	56/–/–	4/0/100	3/0/100	3/2.5/–	16/–		25/29	28/13	16/1/94/1/94/–/–	
		18	48/12/10	3/0/100	3/0/100	0/0/0	6/11		28/29	20/8	12/0/100/0/100/0/100	
		19	36/18/–	3/0/100	3/0/100	1/1/1	16/17		27/27	12/4	5/0/100/0/100/–/–	
		20	46/23.5/–	2/1/50	2/0/100	0/1/0	12/6		25/27	18/11	8/1/88/2.5/69/–/–	
		21	56/–/–	3/1/67	2.5/0/100	0/0/0	–/–		27/28	22/9	12/2/83/3.5/71/3.5/71	
Mean			50.9/19.8/17	3/0.3/89 %	2.7/0.3/93 %	0.5/0.6/0.3	10.1/7.4		27.1/28.6	20.2/8.1	11/0.6/95 %/1.1/90 %/1.2/90 %	
SD±			8.8/4.6/9.8	0.6/0.4/–	0.9/0.8/–	0.9/0.9/0.6	6.3/6.5		2.5/1.5	4.6/4.3	3.1/0.7/–/1.5/–/1.4/–	

MoCA scores pre- and post-procedure show no cognition losses. HADS scores show stable toward improved emotional state (higher scores indicating more emotional distress)

We then set a limit with a preoperative HF32 score more than 28 points over 32, determining a group 1 (*n* = 7, HF32 > 28/32) and a group 2 (*n* = 14, HF32 ≤ 28/32). The preoperative HF32 did not pass Levene’s test for equality of variance as well as all postoperative HF16 scores (2 days, 3 months, and 1 year) and the postoperative handwriting scores, whereas the preoperative ETRS and HF16 scores did. This supports at statistical level the separation of groups 1 and 2 as different patient populations on the basis of the preoperative HF32 score. There are age differences between the two groups, which however did not qualify statistically as separator (mean age was 76.4 ± 4.7 in group 1 and 65.5 ± 8.8 in group 2).

The baseline HF16 was 12.4 (SD± 3.3) in both groups, 15.3 (SD± 1.3) in group 1 and 11.0 (SD± 3.1) in group 2. The mean improvement in HF16 at 3 months for both groups was 74 % (41 % for group 1, 90 % for group 2) and 78 % at 1-year follow-up (40 % in group 1 and 90 % for group 2). The two patients in group 1 treated bilaterally showed 75 and 88 % improvement of HF16 in their dominant hand, as well as 78 % and 56 improvement of HF16 in their non-dominant hand 1 year after the treatment of the second side. There was a highly significant effect of CTT on the HF16 score at 2 days, 3 months, and 1 year post-procedure (*p* < 0.001).

The mean global tremor relief as estimated by the patients was 92 % for the treated hand at 2 days (*n* = 21) and 77 % at 1 year (*n* = 12). Head tremor was never the dominant tremor component and was found in six patients with a mean of 1.2 (SD± 0.57) (*n* = 6) at baseline and 0.25 (SD± 0.2) (*n* = 6) at 3 months. Preoperative postural tremor was found in 17/21 patients (mean 1.7, SD± 1.2). At the latest follow-up, only one patient showed a postural tremor (1–2 over 4) in the treated upper extremity.

### Adverse events

No bleeding occurred. A worsening of pre-existing gait instability was found in five patients, with maximal worsening of 1 point over 4 (mean 0.7/4 ± 0.3). At the last follow-up (3 months to 1 year), only one patient did not fully recover to his original walking ability and was 0.5 points worse than preoperatively. There were no other side effects.

### Targeting precision

**Fig. 3:**
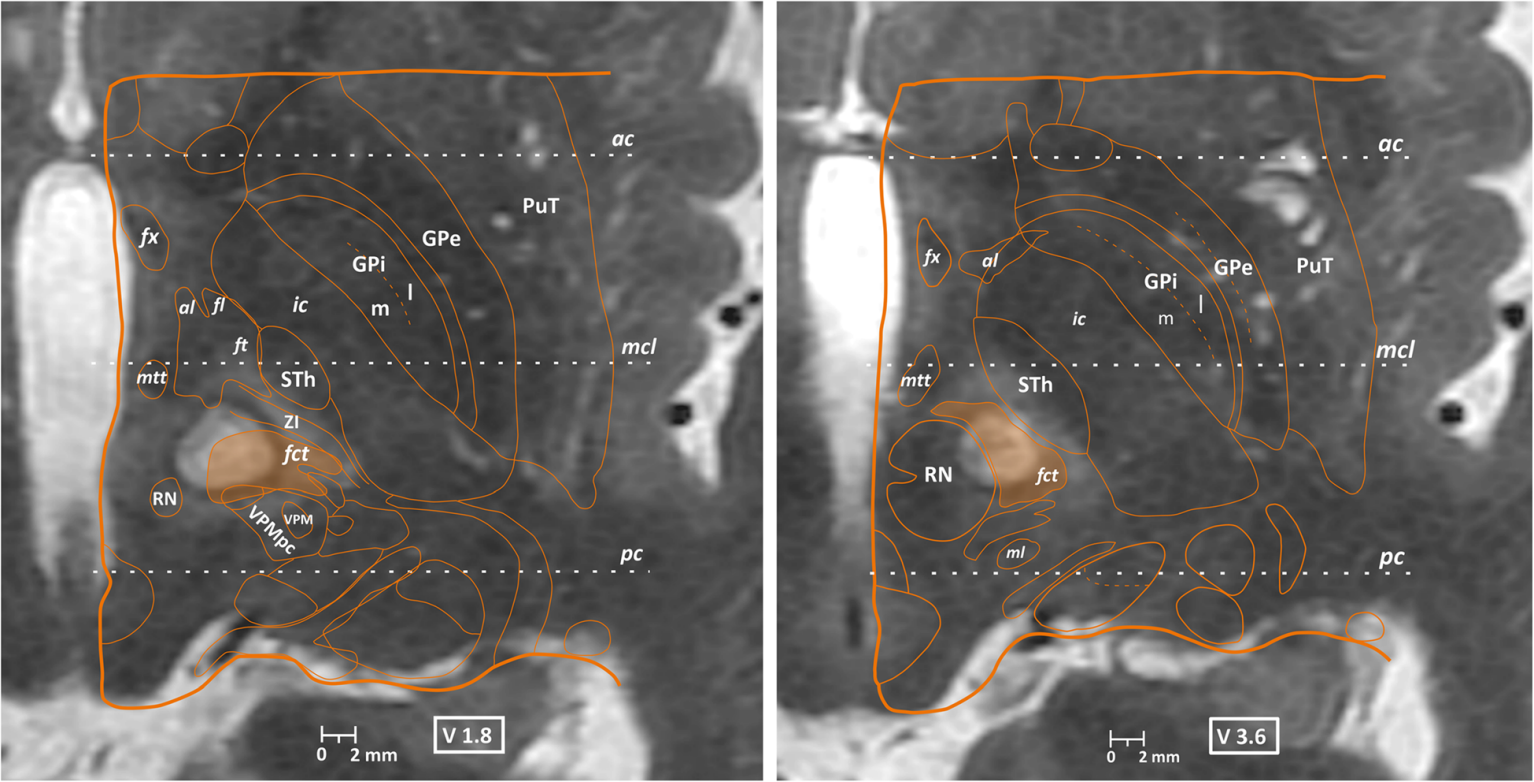
Cerebellothalamic tractotomy on MR scans and atlas maps. MR axial scans taken at 2 days after treatment of patient 19. Atlas maps reproduced from the stereotactic multiarchitectonic Morel *Atlas of the Human Thalamus and Basal Ganglia* superimposed on the ventral 2-mm (*left*) and ventral 4-mm (*right*) axial scans (2, resp. 4 mm below the intercommissural plane). *Orange surfaces* depict the target, the cerebellothalamic tract (fct, for “fasciculus cerebello-thalamicus”) covered by the thermal lesion

**Fig. 4:**
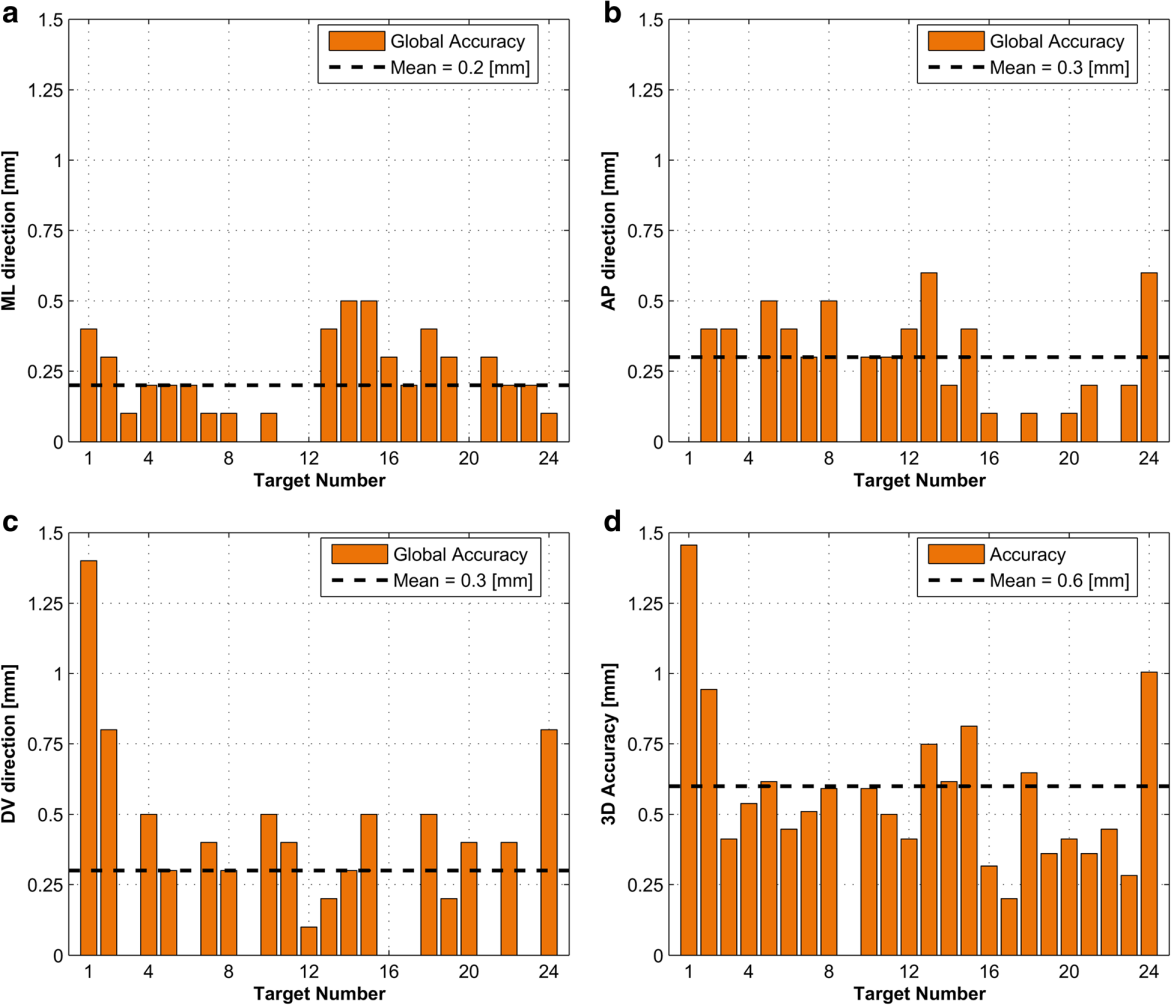
Absolute global targeting accuracy. **a** Mediolateral (ML) direction. **b** Anteroposterior (AP) direction. **c** Dorsoventral (DV) direction. **d** Three-dimensional vector length. The *black dashed lines* show the mean of the accuracies of the 24 realized targets, in each direction and in 3D

Figure 3 shows an example of a CTT lesion on the “V2” and “V4” planes (2, respectively 4 mm under the intercommissural plane) with superimposition of the corresponding maps from the Morel atlas [14]. Figure 4 shows the targeting accuracy values of all performed CTT lesions, with a mean absolute accuracy of 0.2 mm in the ML direction, 0.3 mm in the AP direction, and 0.3 mm in the DV direction. The mean 3D vector accuracy was 0.6 mm.

The applied target reconstruction procedure has of course a given measurement inaccuracy. Error sources, each around half a millimeter, are 1) variations of thalamo-ventricular border position due to differences of MR picture windowing, 2) thickness of the MR slices, 3) determination of the centers of the ac and pc, 4) determination of the center of more or less regular ellipsoidal lesions and 5) variable amounts of lesional and perilesional edema.

## Discussion

### Results

The results presented here demonstrate a postoperative reduction of the ETRS score comparable to other studies. The mean baseline tremor score was also comparable to others (57.6 ± 13.2/144 versus 49.4/144 ± 15.3 for Blomstedt et al. [36], 56.8 ± 12.7 in Nazzaro [60] and 54.9/160 ± 14.4 in Elias [6]). As discussed in the “Results” section, we present separately the spirals, pre- and postoperative handwritings (item 10 of ETRS), and the HF16, after having separated our patients in groups 1 and 2.

Group 1 comprises patients with severe tremor forms (HF32 > 28/32). To our knowledge, the issue of their postsurgical outcome has not been specifically addressed in the literature. Regression analysis in Blomstedt et al. pointed to severity of tremor before surgery as an important predicting factor for residual tremor. In some group 1 patients, we observed, after a good initial evolution, a recurrent pattern of low-frequency (about 1–2 Hz) dysmetric activity on the operated side disturbing drawing, drinking, and handwriting. These tests were scored up to 4 over 4 but do not indicate exclusively the presence of tremor. In an analysis of our group 2 patients, with at least one item at 3/4 but a total functional hand score of ≤8/32, HF16 score improvement was 90 % (*n* = 14) at 3 months and 90 % at (*n* = 9) 1 year. This selected, clearly less affected patient group remains perfectly comparable to other studies (mean ETRS 50.9 ± 8.8 and mean dominant hand score of 11.0/16 ± 3.1 versus 49.4 ± 15.3 and 10.6/16 ± 4.1 for Blomstedt et al. [36]). The evolution of patients 1 and 2 in group 1 provides evidence for two potential additional factors explaining the resistance of tremor in group 1 patients: they showed strong tremor relief after the second bilateral focused ultrasound treatment comprising a target complementation of the first CTT. This points to a possible deleterious effect of a still overactive untreated hemisphere on the other treated side. In addition, the CTT complementation may indicate the necessity of complete target coverage in the context of a particular strong physiopathology. Thus, patients with severe bilateral ET would need a complete and bi-hemispheric CTT treatment. In addition, some of them display an outspoken telo-diencephalic angiopathic state (status cribrosus) which may have been the cause of a reduced response to sonication.

Bilateral treatment has been performed in three patients, with 1-year delay in each patient between the two sides. There is high evidence through the literature advising against bilateral lesioning in the VLp. Our preliminary results show good tolerance for bilateral subthalamic lesioning of the cerebellothalamic tract, as CTT procedures did not produce side effects apart from a slight worsening (increase of 0.5/4) of pre-existent gait instability, noted in 5 over 21 cases. Only one persisted at 1-year follow-up (2.5/4 compared to 2/4 preoperatively). This specific patient had concomitantly a documented polyneuropathy and a cervical canal stenosis which may well have played a role in this persisting slight gait instability.

### Comparison of techniques

There are currently four main surgical options in the treatment of essential tremor: DBS, radiofrequency (RF) lesioning, gamma knife, and newly MRgFUS.

RF lesioning in the VLp is based on a long experience which has shown efficacy and stability over time [17, 19, 20, 22, 23, 26, 61–63]. The rates of neurological deficits in VLp RF lesioning led most of the neurosurgical groups to change their approach over time in favor of DBS. To the present day, VLp RF lesioning is still used in selected situations [64, 65]. Preliminary results of unilateral VLp thalamotomy with focused ultrasound [6, 50, 52] did not show the same extent of adverse events as in RF series, probably due to better lesion control and the incisionless approach. The most common side effects in Elias et al. [6] were paresthesias.

The DBS approach is currently the most frequently chosen (or at least published) option with high rates of tremor control [5, 27–31, 62, 66–69]. The rate of neurological and hardware-related complications of DBS interventions is however significant in the short and long terms [50, 67, 70–75]. As a typical example from a detailed study, Pahwa et al. [66] reported 63 % of dysarthria, 38 % of incoordination, 25 % of paresthesia, and 25 % abnormal gait in bilateral chronic VLp stimulation for ET as well as paresthesias in 56 %, incoordination and dysarthria in 17 % in unilateral stimulation.

Gamma knife thalamotomy has been shown to achieve improvement for tremor scores over the long term averaging 50 to 60 % and low rates of permanent neurological deficits [76, 77], which were mostly due to unpredictable excessive radiation reactions. Bilateral treatment did not show any increase in adverse events with this approach [77]. So far, the main advantage of ultrasound technology over gamma knife, apart from using non-ionizing energy, is the direct control over the lesioning process provided by on-line MR thermometry, reversibility at low temperatures, and direct clinical feedback.

## Conclusions

Our results profile the targeting of the cerebellothalamic tract in the posterior subthalamic area with focused ultrasound as an efficient, minimally invasive, safe, and hardware-free approach against chronic, therapy-refractory essential tremor, combining neurological function sparing with precise targeting and with the possibility to treat bilaterally.

Beyond the option for a technique modality, which should provide maximal safety and precision, the choice of a target should be based on sound physiopathological evidence. In our opinion, the sparing of the thalamocortical network, in this case its motor part, correlates with reduced neurological deficits and thus the possibility to treat bilaterally. This is the primary issue, which has been addressed first in the sixties with RF subthalamotomies [7, 9, 11] as well as nowadays with PSA DBS [33–45] and MRgFUS (this study). This demonstrates that the historical search for a reduction or suppression of deficits can be solved by a proper target choice and not by a given technology. Recent developments tend to address mostly the technical point of view but not the target choice.

The issue of the severe ET patients who are poor responders to surgery remains to be elucidated by further studies. In those patients, bilateral treatment and complete target coverage seem to be necessary in order to achieve satisfactory tremor relief.
